# Classification of medicines and materials in hospital inventory management: a multi-criteria analysis

**DOI:** 10.1186/s12911-022-02069-0

**Published:** 2022-12-08

**Authors:** Amanda G. de Assis, Ana Flávia A. dos Santos, Lucas A. dos Santos, João F. da Costa, Marco Antonio L. Cabral, Ricardo P. de Souza

**Affiliations:** 1 Technology Centre, Federal University of Rio Grande do Norte, Natal, RN Brazil; 2 Centre for Applied Social Sciences, Federal University of Rio Grande do Norte, Natal, RN Brazil

**Keywords:** Multi-criteria decision analysis, FITradeoff, Hospital management, Inventory management, Medicine stock

## Abstract

**Background:**

In the hospital environment, to achieve an optimum level of operations and service, it is necessary to develop adequate inventory management system. Stocks can be managed, amongst other ways, through inputs classification, which is generally carried out based on a single criterion, such as monetary value, demand or criticality, which does not fully address the complexity of a hospital’s inventory management system. Thus, the present study proposes a multi-criteria decision support model to help classify the stock of medicines and materials, enabling a more effective inventory management system for hospitals.

**Methods:**

Methodologically, the study followed 3 stages: (1) preliminary phase; (2) modelling and choice phase; and (3) finalization phase. Each stage had a set of specific steps that were followed. The first stage identified the actors of the process, objectives, criteria and alternatives, establishing 5 criteria and 48 alternatives; the second stage was the choice and execution of the multi-criteria decision method to solve the problem. It was decided to use the Flexible and Interactive Tradeoff method for the sorting problematic. Finally, in the third stage, the sensitivity analysis for the developed model and the validation of the results with decision makers were carried out. In the study, 48 medicines and materials were included to validate the proposed model; however, the model could be used for more items.

**Results:**

From the total of 48 medicines and hospital medical materials selected for the study, the classification of 34 of these alternatives to a single class was obtained through modelling and the other 14 alternatives were destined to two possible classes; moreover, the sensitivity analysis performed showed robust results. The items classified in class W should receive special attention by the stock manager; therefore, they should be monitored weekly. Items classified in class B should be monitored biweekly and finally, items classified in class M, should be monitored monthly.

**Conclusions:**

The classification of medicines and materials developed according to the inventory demands allowed more efficient purchasing decisions, optimizing the stock of materials and medicines at the hospital while optimizing the inventory manager’s activities, saving time. Consequently, the proposed model can support the development of other multicriteria models in different hospital scenarios.

## Introduction

Inventory is composed of materials (either finished or unfinished products) that are under the organization’s possession, in order to address the organizational needs [1]. All organizations keep, in different levels, resources in their inventory, managing them according to their needs; the key difference is the types of materials that are stored in each organization. Inventory management is necessary mainly due to the differences in rhythm between supply and demand [2]. In the health area, inventory can be composed of a variety of materials and medicines with different inflows and outflows, which requires professionals committed to improve the management of resources, processes and activities in order to achieve optimum results [3]. Pharmaceutical products require particular attention as they may compose up to 40% of the healthcare budget. Consequently, inadequate management of the flow of medicines prevents timely access to these items, which in turn results in potential waste of materials as well as risks to patients’ health [4].

With regards to the hospital environment, the purchasing process and inventory management are vital activities; since it is rather difficult predicting their daily consumption. Due to such uncertainty, effective management systems that present solutions to deal with this problem are in high demand [5, 6].

Inventory management optimises cost reductions by promoting the efficient use of the organization’s internal resources [7]. The main challenges refer to planning and control so that adequate levels of each item are dimensioned according to demand [1]. One of the support strategies is the discrimination of the stocked products by degree of importance, allowing for adequate decisions in planning and control [2]. One of the most known methodologies is the ABC classification. The use of the ABC curve is rather beneficial, for it reduces immobilization in inventories without affecting safety, as it controls the items with a varying level of strictness, depending on the item’s importance and demand [8].

Overall, this technique prioritizes items based on the annual value of inventory, which makes this technique evaluate items in terms of contribution to the available budget. However, it is possible that certain drugs, albeit not belonging to category A of the ABC curve, still require considerable attention from the point of view of inventory management because they are critical to the flow of hospital services [9].

Thus, such items are at risk of being neglected as the ABC technique is not sensitive to how essential the drug or material is to the stock. Consequently, some existing techniques incorporate such specificities and other aspects relevant to inventory management; for instance, Vital, Essential and Desirable (VED), which basically considers how essential a certain item is to the stock, but does not consider the cost nor the physical volume that the items will demand.

Another technique for inventory management reported in the literature is called Fast-moving, Slow-moving and non-moving (FSN) in which items are categorized, amongst other factors, as to the frequency with which they are consumed, considering the frequency of items demands, without considering other factors, such as inventory value, criticality and cost. Finally, the technique called Scarce, Difficult and Easily (SDE) aims to manage inventories with a focus on the availability of inventory items on the market, not considering other factors mentioned above [10].

Although each inventory management technique has specific advantages when considering different factors vital to inventory management, it is clear that none of them addresses all these aspects simultaneously. Thus, approaching the problem of inventory management from a single-criteria perspective, despite being valid, may not adequately represent the problem because it does not address the multiple concerns or points of view that the inventory managers must consider in their course of work.

Furthermore, single-criteria approaches fail to incorporate information on individuals’ preferences in relation to medicines and materials with different impacts on the inventory. The preference structure of the manager (decision maker) is considered in the multi-criteria decision analysis methods, as each hospital inventory manager has a different perception of their needs.

In this context the multi-criteria approach to the inventory management problem proves to be advantageous in comparison to the previously described techniques: firstly, for being able to simultaneously evaluate multiple attributes of the items in stock and, secondly, for being an approach that, once designed to support a given decision maker or group, it is capable of including criteria beyond those conventionally considered in inventory management techniques, which highlights the potential of MCDA to elicit and address all factors considered relevant to the decision problem, in this case, the problem of sorting items in stock. Thus, this approach is advantageous as it is able to consider all relevant aspects in an integrated manner, rather than in isolation, as it is the case with single-criteria techniques [11].

The VED, FSN, SDE techniques as well as the ABC Curve are techniques that basically use only a single criterion for stock optimization. Currently, the classification of items is based on only one criterion, which usually in inventories, refers to cost. When working with many items, however, there may be other criteria that represent important management considerations [12]

Supply certainty, obsolescence rate, and the impact of defective items are examples of such considerations. And, in certain cases, some of these may be more important when compared to the cost of items [13].

Thus, due to the need to evaluate two or more decision parameters, the potential of Multi-criteria Decision Analysis (MCDA) methodologies arises, which include several principles and analytical methods for decision making in complex environments [14].

The multi-criteria methodology is a way of approaching complex problems that are characterized by an amalgamation of objectives that sometimes cannot be quantified by measurement or estimation. The multi-criteria approach allows for greater knowledge about the problem and proposed solutions, emphasizing the decision makers’ perspective and judgement, supporting both subjectivity and the objectivity inherent in the decision-making process [15]. Thus, the multi-criteria inventory sorting problematic attracts more and more attention from researchers and becomes the focus of research in inventory management [16].

In hospitals, the inventory manager has to make decisions at all times in order to optimize the stock flow. Thus, the present research aimed to develop a multicriteria decision model to support the decision maker with the classification of materials and medicines for hospital inventory management.

FITradeoff was the multi-criteria decision analysis method chosen for the construction of the model, since it requires less effort on the part of the decision maker for it does not require the determination of the exact indifferent points and allows the use of partial information to obtain the final weight space [17]. Furthermore, the choice of method also occurred because the compensatory rationality of the decision maker was verified in the problem structuring, which recommends additive methods. Moreover, the chosen method works with linear programming, which facilitates the development of the model [18].

Finally, the research also intends to fill a gap found in the systematic review by Assis et al. [11], in which the authors describe articles that address inventory problems involving multi-criteria. It was identified that 38.3% of the applications of the articles reviewed were based on numerical experiments or data replication, which ends up generating a lack for real applications linked with decision makers.

## Multi-criteria decision analysis

Decision-making is extremely intuitive when considering single-criteria problems, as it only requires choosing the alternative with the highest preference rating [19]. But when considering different choices or courses of action, the problem turns into multi-criteria decision-making (MCDM) or multi-criteria decision analysis (MDCA) [17]. The main objective of multi-criteria decision analysis is to facilitate the decision makers’ understanding about the problem faced, their own organizational priorities, values and goals, besides offering guidance in identifying a preferred course of action [11, 20, 21]. The MCDA for problem classification is one of the critical issues in all kinds of operations management and can help organizations because it is possible to validate the generated decision rules with multiple strategies [22].

### FITradeoff method for problem classification

The FITradeoff method is based on the traditional Tradeoff method, although the procedure based on tradeoffs has a robust axiomatic structure, the definition of the weights used in the additive model is achieved from exact values of indifference to the consequences defined by the decision maker [17, 23]. The study by Weber and Borcherding [24] claims that this form of weight elicitation requires high cognitive effort, presenting the decision maker’s answers with 67% of inconsistencies.

Thus, FITradeoff (Flexible and Interactive Tradeoff) appears with the objective of obtaining a flexible elicitation process, which requires less effort from the decision maker and, in addition, the decision maker does not need to make adjustments for the indifference between two consequences, which, in turn, leads to less inconsistency during the preference survey process [17].

FITradeoff for problem classification establishes classes or categories for the problem using lower and upper bounds denoted by *br*. The decision maker determines these values that must be between 0 and 1, and represent, respectively, the worst and the best global $v(a_{j} )$ of a given alternative [23].

Therefore, two Linear Programming Problem (LPP) are solved for each alternative $a_{j}$ belonging to the discrete set of alternatives within the problem, in order to calculate the maximized and minimized global values for each alternative (Eqs. 1–14). Considering the current space of the $\varphi^{n}$ weights obtained from the information that the decision maker has provided. As the decision maker provides more preferred information throughout the process, the weight space is refined and the differences are updated [25].

#### LPP 1

1 $$s_{1} = Min_{{k \in \varphi^{n} }} v\left( {a_{j} } \right) = \mathop \sum \limits_{i = 1}^{n} k_{i} v_{i} \left( {a_{j} } \right)$$

s.t.

2 $$v_{1} \left( {X_{1}^{\prime \prime } } \right)k_{1} + \varepsilon \le k_{2}$$

3 $$v_{1} \left( {X_{1}^{\prime } } \right)k_{1} - \varepsilon \ge k_{2}$$

$$\vdots$$

4 $$v_{n - 1} \left( {X_{n - 1}^{\prime \prime } } \right)k_{n - 1} + \varepsilon \le k_{n}$$

5 $$v_{n - 1} \left( {X_{n - 1}^{\prime } } \right)k_{n - 1} - \varepsilon \ge k_{n}$$

6 $$\mathop \sum \limits_{i = 1}^{n} ki = 1$$

7 $$k_{i} \ge 0,\quad i = 1, \ldots ,n.$$

#### LPP 2

8 $$s_{2} = Max_{{k \in \varphi^{n} }} v\left( {a_{j} } \right) = \mathop \sum \limits_{i = 1}^{n} k_{i} v_{i} \left( {a_{j} } \right)$$

s.t.

9 $$v_{1} \left( {X_{1}^{\prime \prime } } \right)k_{1} + \varepsilon \le k_{2}$$

10 $$v_{1} \left( {X_{1}^{\prime } } \right)k_{1} - \varepsilon \ge k_{2}$$

$$\vdots$$

11 $$v_{n - 1} \left( {X_{n - 1}^{\prime \prime } } \right)k_{n - 1} + \varepsilon \le k_{n}$$

12 $$v_{n - 1} \left( {X_{n - 1}^{\prime } } \right)k_{n - 1} - \varepsilon \ge k_{n}$$

13 $$\mathop \sum \limits_{i = 1}^{n} k_{i} = 1$$

14 $$k_{i} \ge 0,\quad i = 1, \ldots ,n.$$

In $s_{1}$(1) and $s_{2}$(8) are the optimal solutions of LPP 1 and LPP 2, respectively, and ε is a constant used to make strict differences computationally tractable. The Eqs. (2–7) and the Eqs. (8–14) are constraints of LPP 1 and LPP 2, respectively.

Furthermore, after obtaining the maximum and minimum values $v(a_{j} )$ of the problems, another rule is applied, in order to decide whether or not each alternative $a_{j}$ will be assigned to a category [25].

According to the current weight space it is not possible to assign *a*_*j*_ to a single category.
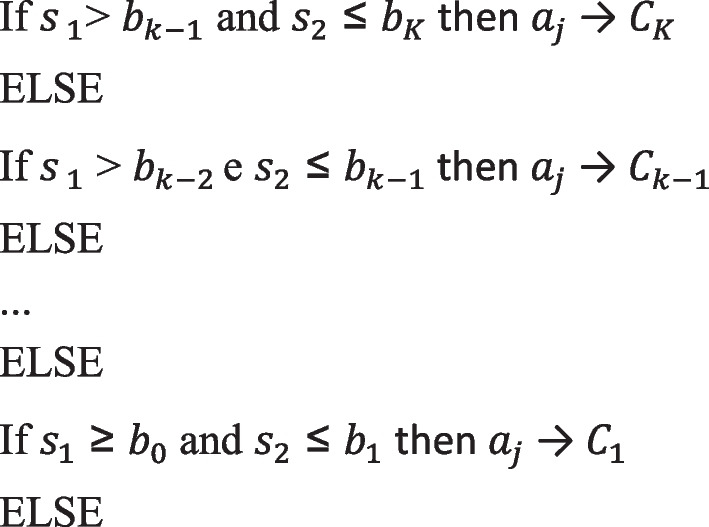


During the flexible elicitation process, each time the decision maker answers a question, the weight space is updated, changing some of the constraints presented in linear programming problems (LPP 1) and (LPP 2). When all alternatives are classified into single categories, or when the decision maker is no longer willing to continue the elicitation process, or when the partial results obtained in a given weight space are considered sufficient to support the decision, the process is concluded. [26].

Finally, in the multi-criteria model developed, each criterion will have an associated weight range to classify the materials and medicines in the inventory. It is noteworthy here that each inventory manager may have a different view and the weights of each criterion can be adjusted.

## Methods

For the development of a multicriteria model, the procedure proposed by Almeida [23] was adopted, which is presented on Table 1.

**Table 1:** Problem structuring phases

Preliminary phase	Modelling phase	Finalisation phase
1. Characterize decision-maker(s) and other actors 2. Identify goals 3. Establish criteria 4. Establish space for actions and problems 5. Identify uncontrolled factors	6. Design the preference modelling 7. Carry out an intra-criteria assessment 8. Carry out an inter-criteria assessment	9. Evaluate alternatives 10. Carry out sensitivity analysis 11. Analyse results and make recommendations 12. Implementing the decision

### Preliminary phase

As the research focused on the materials and medicine inventory in a hospital in Brazil—HUOL, the actors selected for the construction of the model were: (1) the decision-maker (inventory manager) who effectively participates in the construction of the model by establishing criteria and validating data in the elicitation and decision-making process; (2) the specialist (inventory management assistant) who collaborates with the collection and validation of information; and (3) the analysts (researchers) who participate in all stages so that the model is built satisfactorily. They were chosen for the research due to the knowledge they have about how inventory management works, in addition to the experience that the manager possesses as a pharmacist.

At the end of the execution of the decision model the following objectives were established:

- *Effective budget control* To ensure that financial resources are optimally distributed amongst the existing items in the inventory, respecting institutional limits.
- *Hospital care needs* To secure essential material or medication prompt availability upon request.
- *Operational efficiency* To ensure adequate resupplying schedules.
- *Inventory security* To guarantee that the most critical items in the stock do not run out.
- *Structural adaptation* To balance the assistance service´s needs with the availability of physical space.

The criteria were described and determined according to the objectives presented and are displayed in Table 2.

**Table 2:** Characterization of the criteria evaluated

Criteria	Related objective	Description	Attribute type	Function type
Cost	Balancing budget control	Unit cost of each item in Brazilian Reais (R$)	Natural	Minimization
Demand	Meet inventory demand	Monthly demand of each item in unit	Natural	Maximization
Lead time	Ensuring stock replenishment	Time (in days) from supplier confirmation until the item is available for use in stock	Natural	Minimization
Volume	Work with available physical space	Dimension of space (in m^3^) that the item occupies	Natural	Minimization
Criticality	Avoid shortage of critical items	Valuation of the item according to its need in the hospital—in 1 (low), 2 (medium) or 3 (high)	Built	Maximization

Criticality was constructed on a 3-level scale and the clarity of each level was based on the ABC-VED-FNS criticality rating that Gizaw and Jemal [9] proposed to categorize items in the Pharmaceuticals Inventory Management. Considerations about the definitions of this scale can be found on Table 3.

**Table 3:** Criticality scaling

Criticality level	Significance	Description	Inventory manager attention
W (weekly)	High criticality	If the item is missing, it can cause downtime and jeopardize the safety of the patient and institution; it cannot be replaced by other equivalents	The inventory manager must monitor weekly for items classified in this class
B (biweekly)	Medium criticality	If the item is missing, it can cause downtime and put people and the environment at risk; it can be replaced with relative ease	The inventory manager must monitor biweekly for items classified in this class
M (monthly)	Low criticality	If the item is missing, it does not cause downtime or risks to patient safety; it can be easily replaced and is also easy to procure	The inventory manager must monitor monthly for items classified in this class

For the multicriteria model developed validation, 48 inventory items were selected, referring to 26 medicines and 22 medical and hospital supplies (materials), whose performance information for each of the criteria under analysis was available.

These 48 items were selected according to the experience of the inventory managers and their team, because after running the model developed, they should analyse whether they really agreed with the class in which the items were inserted.

The sorting problematic [18, 25] guided the construction of the decision model. This approach was chosen in order to recommend a classification procedure similar to the ABC, VED, FSN, SDE technique, which meets the decision maker’s preferences, but also considers multi-criteria simultaneously.

It is expected that the items allocated to **class W** will be those with greater focus on inventory management, since it is not feasible to allocate equal efforts to all items.

### Modelling stage

It was observed that the decision maker’s preference structure incorporates strict preference and indifference relations. Thus, it was defined that the structure (P, I) is the one that describes the decision maker’s preference behaviour in the face of comparisons between two alternatives, in which *P* describes the strict preference relation in favour of one of the alternatives and *I* designate the binary relation of indifference in which the two actions are judged as equivalent.

The decision maker’s rationality proved to be compensatory, as there is compensatory balance between the criteria presented, which means that a disadvantage in one criterion can be balanced against an advantage in another criterion. For instance, losses in the cost criterion can be balanced by gains in the criticality criterion.

Based on the described particularities, the FITradeoff method was designated as it operates with the structure (*P, I*) and considers compensatory rationality. Furthermore, as it is a partial information method, it is expected that less cognitive effort will be required from the decision maker to declare preference information [18, 23].

Before the next steps in the FITradeoff for sorting problematics, it was necessary to elicit one more variable, referring to the limits of the classes, which culminated in the definition with the decision maker of the limits of three classes, established by Class W, Class B and Class M, in such a way that W > B > M. The threshold values that define the boundaries of the class established by the decision maker were $b_{1}$ = 0.4 and $b_{2}$ = 0.7 all these thresholds are listed in Table 4.

**Table 4:** Limit value range for each category

Class **W**	0.7 < *v*(*a*_*j*_) ≤ 1
Class **B**	0.4 < *v*(*a*_*j*_) ≤ 0.7
Class **M**	0.0 ≤ *v*(*a*_*j*_) ≤ 0.4

The limits of each class can be adjusted by decision makers, as the model developed can be adapted to different realities. By adjusting these limits, the number of items in each class will change and, consequently, the frequency with which the inventory manager will monitor the item too.

In this application and validation of the developed multicriteria model, the decision maker determines the limits in order to be proportional to all classes.

The last steps of the modelling phase, which are intra-criteria/inter-criteria assessment, were performed in the FITradeoff Decision Support System (DSS) environment and will be described in the “Results” section.

The FITradeoff decision support system incorporates mathematical programming of this multicriteria method as proposed by Kang et al. [25] in which the authors adapt the FITradeoff method developed by Almeida et al. [17], for sorting problematics. Like the method originally designed for the problem of choice [17] and, later, for ranking problematics [26], the FITradeoff method for sorting problematics is based on a flexible elicitation process in which partial preference information is sufficient for the alternatives to be allocated to the categories, so that the allocation is consistent with the decision maker’s preference system [14].

This method operates within the scope of multi-attribute value theory (MAVT); thus, interactions between the decision analyst and the evaluator, in support of the DSS FITradeoff, were conducted so that the final recommendation was suggested. The activities listed in Table 1 were performed recursively. The FITradeoff DSS for sorting problematic is a technological solution developed by the Centre for Development in Information and Decision Systems and implements the mathematical formulation of the multi-criteria method used in the current work to facilitate the decision-making process. Further information, including access to the FITradeoff DSS, can be consulted at http://fitradeoff.org/.

**Fig. 1:**
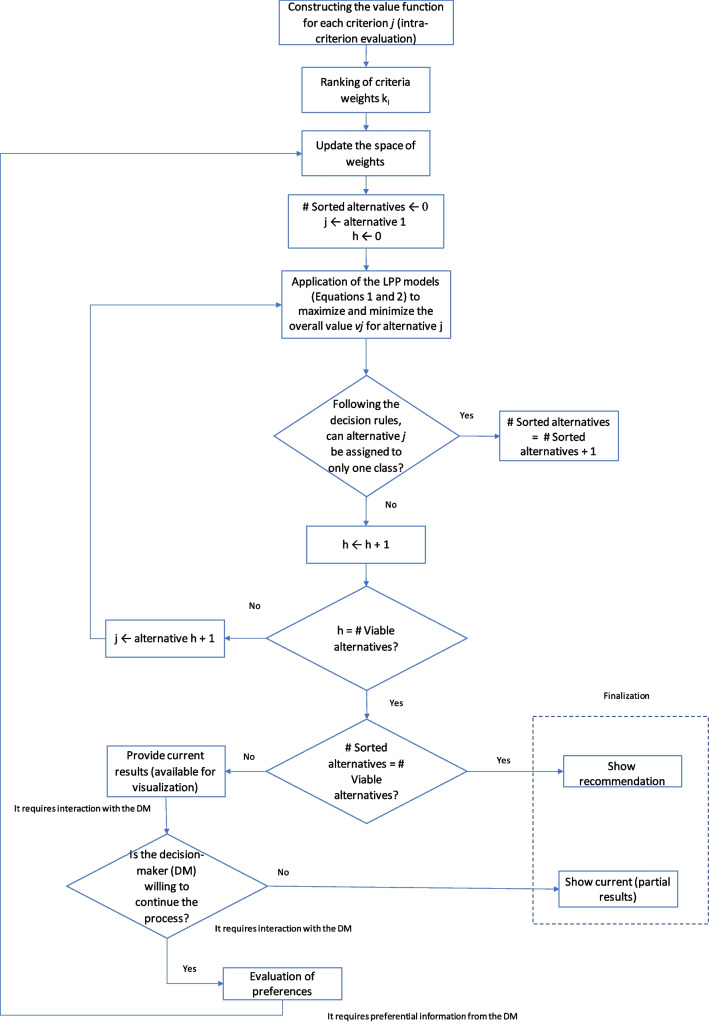
FITradeoff process

Further to the facilitation activities described in Table 1, the process of applying the FITradeoff method followed the flowchart shown in Fig. 1, in which the interaction activities between the facilitator and the decision maker are broken down according to the legend presented. It is important to highlight that the use of FITradeoff DSS covers activities 6 to 10 of Table 1, thus configuring itself as a software to support the methodological execution of the decision model construction.

### Finalization stage

The result analysis stage supports the suggestions proposed by the model developed taking into account the explored decision context and the assumed simplifications. The sensitivity analysis was performed to test the robustness of the results obtained, in which the nominal values of the limits of the classes were varied by ±10% in order to investigate possible variations in the allocation of alternatives to pre-established categories. Finally, with the results and their analysis, it was possible to make recommendations to the decision maker, so that in the model outcome, it can be validated and replicated. All these steps will be explained in the “Results and discussions” sections.

## Results and discussion

A total of 48 items were part of the current study; the values of the criteria: costs, demand and lead time were obtained directly from the hospital’s procurement system. The criticality criteria were constructed based on a 3-level scale and the volume criterion was determined by using a measuring tape for each item.

The consequences matrix and class limits are the input data for the FITradeoff Decision Support System–DSS. Table 5 shows the values of the matrix in which the performance of each alternative in the given criterion is presented.

**Table 5:** Consequence matrix

Alternatives	Cost (R$)	Demand (unit)	Lead time (days)	Criticality	Volume (m^3^)
Alteplase, 50 mg, lyophylic powder	2022.99	5	1	3	0.0004233
Calcium folinate, 50 mg, lyophylic powder	10.72	250	48	2	0.0046800
Cefazoline sodium, 1 g, lyophylic powder	16.28	948	4	2	0.0022298
Ceftriaxone sodium, 1 g, lyophylic powder, indovenous	6.51	1067	20	2	0.0035784
Concentrated enzyme detergent—liter	48.00	83	28	3	0.0196070
Disposal syringe 20 ml without needle—luer lock	0.44	2655	28	3	0.0311555
Double lumen catheter for long permanent HD. 14.5FR × 36 cm	900.00	3	13	2	0.0018360
Double lumen subclavia access catheter—7F × 20CM	69.81	60	9	3	0.0106000
Enoxaparin sodium, 40 mg/0.4 ml, filled syringe	12.92	1529	5	3	0.0329586
Ethyl alcohol 70% P/V—100 ml	1.19	2946	57	2	0.0090137
Ethyl alcohol 70% P/V—FR 250 ml	2.55	2594	43	2	0.0380800
ethyl alcohol, 70%, GE—FR 500 g	7.10	351	11	2	0.0285120
Face protection mask, respirator type—N95/PFF-2	6.46	1047	27	3	0.0038850
Fentanyl citrate, 0.05 mg/ml, injectable solution, 5 ml	1.85	1174	16	2	0.0004620
Glove for non-surgical latex procedure—small size	0.38	23,298	15	2	0.0286202
Glutaraldehyde aqueous solution for general use 2%	190.00	21	36	2	0.0314712
Human albumin, 20%, injectable solution, 50 ml	99.50	476	14	3	0.0065340
Low pressure extender male and female connector—20 cm	4.90	523	18	3	0.0302568
Medium–large clip, for laparoscopic clip	19.79	156	19	3	0.0009570
Meropenem, 1 g, lyophylic powder	20.44	1433	20	3	0.0010129
Methylcellulose, 2%, intraocular solution, 1.5 ml	29.60	120	21	1	0.0002550
Myelogram aspiration needle 16 g—6 cm to 7 cm	60.00	33	7	2	0.0060204
Nalbufine chloridrate, 10 mg/ml, injectable solution, 1 ml	9.28	232	26	3	0.0003931
Natural latex surgical glove-no. 8.0-PAR	1.35	1818	33	3	0.0355320
Oxacillin, 500 mg, lyophylic powder	1.75	2347	12	3	0.0017922
Oxaliplatin, 100 mg, lyophylic powder	90.00	29	14	2	0.0016027
Percutaneous radial introductor with hemostatic valve—5F-11 cm	44.68	34	56	3	0.0058332
Piperacillin sodium + tazobactam sodium (4 g + 500 mg), lyophylic powder	16.24	1268	28	2	0.0018198
Radiographic film, size 35 × 43 cm for laser IMP	4.41	647	28	3	0.0257418
Radiological contrast, non-ionic, low osmolarity, 300 to 320 mg iodine/ml, injectable solution, 100 ml	53.07	527	7	3	0.0003856
Radiological contrast, non-ionic, low osmolarity, based on ioversol, 320 mg of iodine/ml, solution for injection, 125 ml	232.52	45	5	1	0.0267400
Rectangular surgical mask with strips—3 layers	2.32	29,155	57	3	0.0655928
Remifentanil chloridrate, 2 mg, lyophylic powder	36.60	72	15	1	0.0001599
Sevofluran, inhaled anesthetic, 250 ml	295.00	47	10	3	0.0005152
Small clip for laparoscopic clipper	30.00	45	13	3	0.0009660
Sodium chloride, 0.9%, injectable solution, closed system, 100 ml	1.59	11,031	17	3	0.0218919
Stabilizer for coronary surgery with suction mechanism	2042.93	3	18	3	0.0130816
Sterile hydrophyl gaze compress—7.5 cm × 7.5 cm—package with 10 units	0.39	26,007	8	3	0.0461448
Straight scalpel for paracensis (auxiliary incision)—15 degrees	22.00	75	19	3	0.0013440
Surgical glove in natural latex-no. 7.0	1.33	4342	33	3	0.0362952
Surgical glove in natural latex-no. 7.5	1.00	5089	47	3	0.0357840
Surgical trepanning tool for receiver cornea—8.0 mm	360.00	5	20	2	0.0007313
Suventanil citrate, 50 mcg/ml, injectable, 1 ml	20.12	125	13	1	0.0006350
Syringe desc. for insulin 1 ml, with needle	0.49	4339	133	3	0.0381779
Team for administration of parenteral solutions by infusion pump	19.74	1600	28	3	0.0365381
Thyrofibana chloridrate, 0.25 mg/ml, injectable solution, 50 ml	876.00	26	42	3	0.0002125
Tigecycline, 50 mg, lyophylic powder	184.34	48	15	3	0.0003060
Voriconazole, 200 mg, lyophylic powder	148.88	9	16	1	0.0046406

Once the input data has been incorporated, it is necessary, as a first step, the ordering of the criteria scaling constants. Hence, the criteria were organized in descending order of importance according to the decision maker’s preferences, considering the range of consequences of the problem and not the intrinsic importance of the criterion, as follows: K_demand_ > K_criticidad_ > K_lead time_ > K_cost_ > K_volume_. From this ordering, the space of scaling constants is formed.

Therefore, the flexible elicitation process is conducted. In this phase, there is a comparison between pairs of hypothetical consequences with different performances, in which the decision maker must express his preferences. Figure 1 shows the first cycle of the process.

At this moment, the decision maker will make a paired comparison between options.

**Fig. 2:**
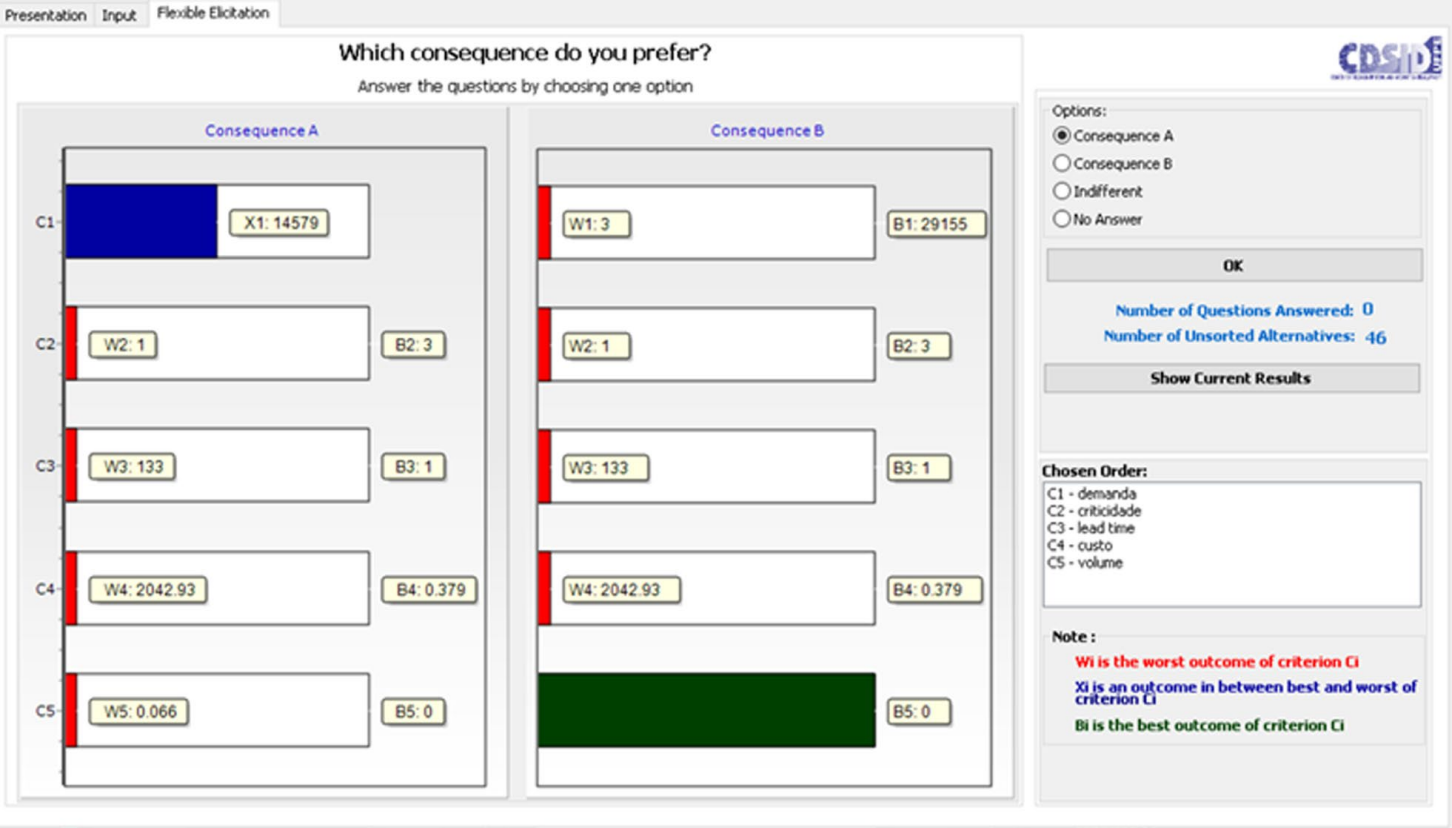
Elicitation process—first cycle

In Fig. 2, the decision maker must choose between having an alternative with 50% performance in criterion 1 or 100% in criterion 5, that is, the decision maker will demonstrate his preference for items that have a demand of 50% in comparison to the others or items that take up a lot of space (volume) in inventory. It is worth noting that 100% in the volume criterion refers to the item that takes up the most space amongst the 48 selected for model validation.

The weight space initially defined only with the constraint of the ordering of the scaling constants is updated as the preference information is declared by the decision maker. The information is obtained based on cycles of comparison between hypothetical alternatives constructed based on the space of consequences (Fig. 1). At the end of the elicitation process, it is expected that each alternative, in this case referred to by each of the 48 stock items under evaluation, will be assigned to a single class. Table 6 provides a summary of the elicitation process.

**Table 6:** Summary of the elicitation process

Number of cycles	Preference among consequences	Number of classified alternatives
0		2
1	A	2
2	B	6
3	B	7
4	A	10
5	A	10
6	B	10
7	B	10
8	B	17
9	B	17
10	B	17
11	B	33
12	B	33
13	B	33
14	B	33
15	A	33
16	B	33
17	A	34
18	A	34
19	A	34
20	A	34

Table 6 informs the outcomes of each facilitator/decision maker interaction during the elicitation process, in order to define the values of the variables to apply the additive model. The FITradeoff method was used to support the elicitation of the values of the criteria scale constants ($k_{i}$) in which, through an iterative and flexible process, conducted with the support of the FITradeoff Decision Support System–DSS, only partial information from the decision maker was required. The first iteration represented—cycle 0 in Table 6—is the one resulted after the insertion of the ordering information of the criteria weights in the DSS. Thus, initially LPP1 (Eqs. 1–7) and LPP2 (Eqs. 8–14) are executed for each alternative with the information of the ordering of the weights as constraints.

The first line in Table 6 informs that only with the ordering of the criteria weights, 2 of the 48 alternatives could already be allocated to a single class. Between interactions 1 to 20, with the support of the FITradeoff DSS, the analyst presented the decision maker with pairs of hypothetical alternatives whose performances differed only in two criteria, and requested the decision maker to provide preference information about the pair of alternatives.

The FITradeoff method is compatible with strict preference information or indifference; thus, the decision maker could either inform a preference of one hypothetical alternative over another (strict preference, *P*) or indifference between the two alternatives (*I*). Moreover, in order to promote less cognitive effort to the decision maker, FITradeoff also allows the comparison to be skipped if the decision maker still does not have enough information to compare those two alternatives.

The elicitation process was conducted as described in Fig. 1. In Fig. 2, it is displayed the FITradeoff interface screenshot of cycle 1 paired comparison. Given two hypothetical alternatives A and B, the first with intermediate performance in the “demand” criterion and the worst performance in the others, the second with the best performance in the “volume” criterion and the worst performance in the others, the decision maker is asked to express his preference in relation to this pair of hypothetical alternatives. In this case, the inventory manager confirmed that he preferred alternative A.

From that instance forward, at each preferred information provided up to the last cycle, that is, at each analyst-decision maker interaction, the weight space is updated alongside LPP1 and LPP2 with the current level of information.

In 20 cycles total, 34 alternatives were allocated to a single class while 14 alternatives were suggested to two viable classes. It is worth noting that class W is considered the most preferable and class M is the least preferable. As a practical implication, the items allocated to class W are the most critical to inventory management whilst those allocated to class M are the least critical. Table 7 presents the result of the alternatives classified to a single class.

**Table 7:** Classified alternatives

Alternative	Minimum overall value	Maximum overall value	Class
Alteplase, 50 mg, lyophylic powder	0.41873197	0.48394727	B
Concentrated enzyme detergent—liter	0.42028734	0.56357202	B
Disposible syringe 20 ml without needle—luer lock	0.47157452	0.58919982	B
Disposible syringe for insulin 1 ml, with needle	0.49378278	0.50515474	B
Double lumen subclavia access catheter—7F × 20 cm	0.41982870	0.59089520	B
Enoxaparin sodium, 40 mg/0.4 ml, filled syringe	0.44912142	0.59592977	B
Face protection mask, respirator type—N95/PFF-2	0.43951006	0.59574410	B
Glutaraldehyde aqueous solution for general use 2%	0.20970497	0.39418957	M
Human albumin, 20%, injectable solution, 50 ml	0.42812398	0.59329625	B
Low pressure extender male and female connector—20 cm	0.42906119	0.57208599	B
Medium–large clip, for laparoscopic clip	0.42174300	0.59443520	B
Meropenem, 1 g, lyophylic powder	0.44720712	0.61021685	B
Methylcellulose, 2%, intraocular solution, 1.5 ml	0.00233305	0.31348742	M
Nalbufine chloridrate, 10 mg/ml, injectable solution, 1 ml	0.42325848	0.58929397	B
natural latex surgical glove-no. 8.0-PAR	0.45488425	0.56840435	B
Oxacillin, 500 mg, lyophylic powder	0.46543282	0.63119120	B
Percutaneous radial introductor with hemostatic valve-5F–11 cm	0.41931025	0.54734040	B
Radiographic film, size 35 × 43 cm for laser IMP	0.43153382	0.56771800	B
Radiological contrast, 300 to 320 mg of iodine/ml, injectable solution, 100 ml	0.42914095	0.61045399	B
Radiological contrast, ioversol based, 320 mg of iodine/ml, injectable solution, 125 ml	0.00083750	0.28998712	M
Rectangular surgical mask with strips—3 layers	0.87555622	1.00000000	W
Remifentanil chloridrate, 2 mg, lyophylic powder	0.00137590	0.31882647	M
Sevofluran, inhaled anesthetic, 250 ml	0.41956947	0.58533668	B
Small clip for laparoscopic clipper	0.41952959	0.59862967	B
Stabilizer for coronary surgery with suction mechanism	0.41869209	0.45209266	B
Sterile hydrophyl gaze compress-7.5 cm × 7.5 cm—package with 10 units	0.90501007	0.93722708	W
Straight scalpel for paracensis (auxiliary incision)—15 degrees	0.42012781	0.59283364	B
Surgical glove in natural latex-no. 7.0	0.50521428	0.60111437	B
Surgical glove in natural latex-no. 7.5	0.52010990	0.59678555	B
Suventanil citrate, 50 mcg/ml, injectable, 1 ml	0.00243275	0.32221785	M
Team for administration of parenteral solutions by infusion pump	0.45053720	0.56860837	B
Thyrofibana chloridrate, 0.25 mg/ml, injectable solution, 50 ml	0.41915072	0.51448538	B
Tigecycline, 50 mg, lyophylic powder	0.41958941	0.58735622	B
Voriconazole, 200 mg, lyophylic powder	0.00011964	0.30529318	M

According to the multicriteria model developed, items that were allocated in two classes should be considered in the most critical class, that is, if an item was in classes B and W, the item must be monitored like the other items in class W (weekly).

Medicines and materials assigned to class W, estimated as the most preferable, obtained maximum and minimum global values that ranged from 1 to 0.7; those assigned to class B, presented maximum and minimum *v*(*a*_*j*_) between 0.7 and 0.4; and finally, those allocated to class M, considered less preferable, had values ranging between 0.4 and 0. For instance, the sterile hydrophilic gauze pad presented a minimum and maximum global value, respectively, 0.9050 and 0.9372, which made this alternative to be assigned to the most preferable class, this can be explained by the fact that this item manifests high demand and criticality.

The possibility of classifying into two viable classes occurs when the space of scaling constants updated from the information given by the decision maker and the linear programming problem calculated from this information present global values that do not fit the limits established for a single class. This is to be expected because FITradeoff operates in the context of partial information.

For instance, the aspiration needle had global minimum and maximum values, respectively, 0.2099 and 0.4586, with a minimum value within the parameters of Class M (0.0 ≤ *v*(*a*_*j*_) ≤ 0.4) and the maximum value within the parameters of Class B (0.4 < *v*(*a*_*j*_) ≤ 0.7), which makes categorization into a single class impossible. Table 8 shows the alternatives that were between two possible classes.

**Table 8:** Alternatives between viable classes

Alternative	Minimum overall value	Maximum overall value	Class
Calcium folinate, 50 mg, lyophylic powder	0.21427137	0.42274553	B, M
Cefazoline sodium, 1 g, lyophylic powder	0.22818990	0.48049510	B, M
Ceftriaxone sodium, 1 g, lyophylic powder, indovenous	0.23056283	0.46447774	B, M
Double lumen catheter for long permanent HD. 14.5FR × 36 cm	0.20934604	0.40239618	B, M
Ethyl alcohol 70% P/V-100 ml	0.26803118	0.44529085	B, M
Ethyl alcohol 70% P/V-FR 250 ml	0.26101210	0.42631294	B, M
Ethyl alcohol, 70%, GEL-FR 500G	0.21628537	0.43957048	B, M
Fentanyl citrate, 0.05 mg/ml, injectable solution, 5 ml	0.23269647	0.47352129	B, M
Glove for non-surgical latex procedure—small size	0.67386186	0.73996574	W, B
Myelogram aspiration needle 16 g—6 cm to 7 cm	0.20994426	0.45862365	B, M
Oxaliplatin, 100 mg, lyophylic powder	0.20986450	0.45367685	B, M
Piperacillin sodium + tazobactam sodium (4 g + 500 mg)	0.23457088	0.45983831	B, M
Sodium chloride, 0.9%, injectable solution, closed system, 100 ml	0.63859685	0.72103789	W, B
Surgical trepanning tool for receiver cornea-8.0 mm	0.20938593	0.43064704	B, M

**Fig. 3:**
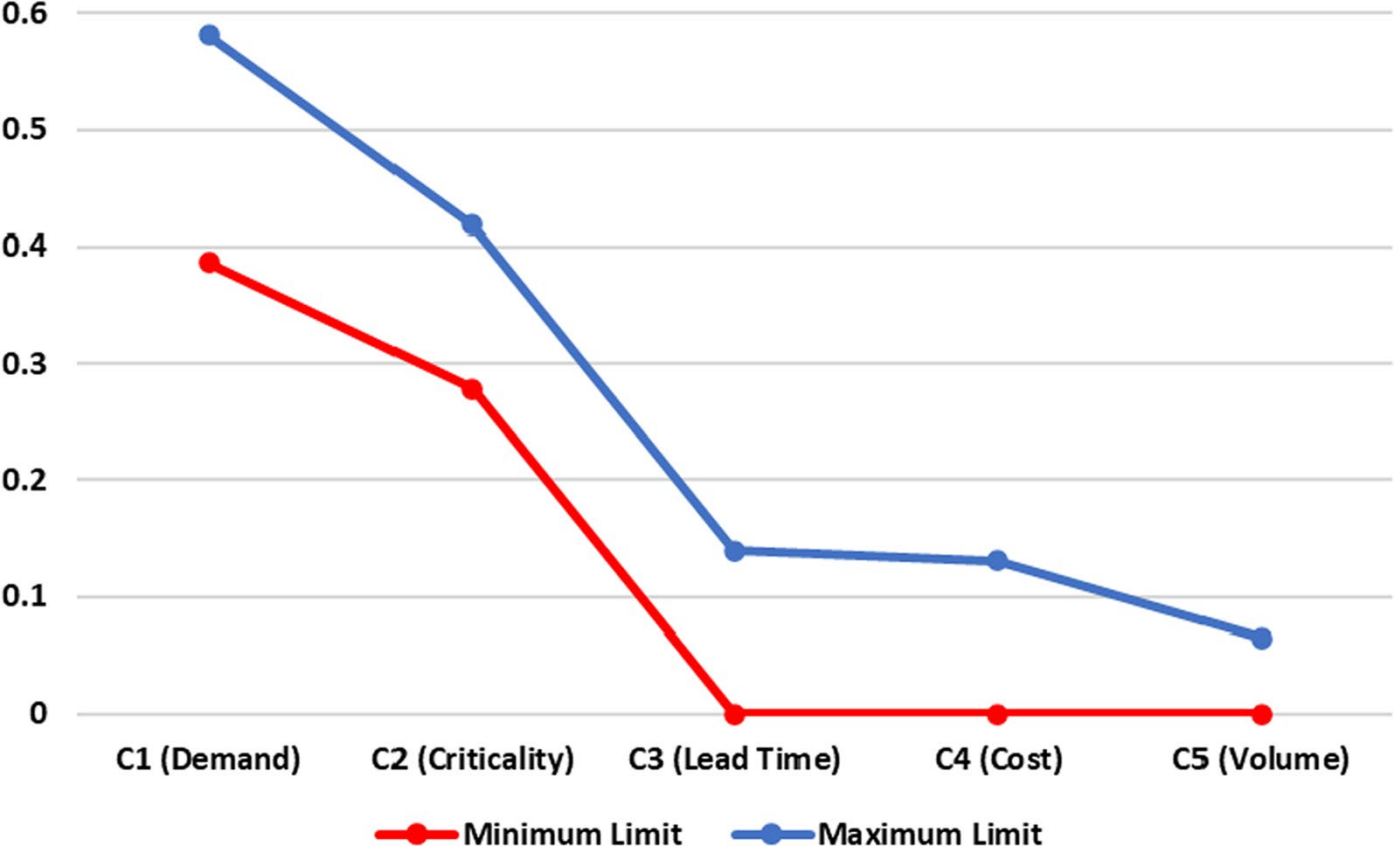
Variation of scaling constants

At the end of the elicitation process, one of the outputs of the Decision Support System is the intervals of the scaling constants of each criterion that defines the final weight space. This assumes that whatever value the constant can take within this range, the classification result will remain the same, as can be seen in Fig. 3, in which C1 represents the demand criterion, C2 criticality, C3 lead time, C4 cost and C5 volume.

A range of weights for each criterion is defined, as this requires less effort from the decision maker. In order to define a weight for each criterion, a lot of effort will be required from the decision maker, which will result in an increase in the number of cycles highlighted in Table 6.

This is a great advantage of the FITradeoff method, as it requires less cognitive effort from the decision maker and, consequently, avoids inconsistency in decisions.

In order to test the robustness of the multi-criteria model developed, variations were performed in the class’s profile nominal values, as performed by Kang [25]. These values are stipulated by the decision maker and define the limits of the class profiles. In the execution of the method, the classes limits defined by the decision maker were respectively $b_{1}$ = 0.4 e $b_{2}$ = 0.7. To verify the performance of the result against these values, a variation of ±10% was determined, so the limits previously established, now in the analysis, can vary between 0.36 ≤ $b_{1}^{\prime }$ ≤ 0.44 e 0.63 ≤ $b_{2}^{\prime }$ ≤ 0.77. As soon as the profile intervals of variation were determined, 10,000 simulation cycles were carried out in which the limits $b_{1}$ and $b_{2}$ were designed randomly.

Sensitivity analysis was performed using a Monte Carlo simulation set in Microsoft Office Excel^®^ with support of the Visual Basic programming language. At each new generated profile limit, the program checked the global maximum and minimum values that were obtained through the FITradeoff method, in order to verify in which class each alternative would be allocated. In sequence, the algorithm verified if there was a difference between the classification of the nominal solution obtained by the method and the one recommended from the simulation. This deviation from the initial ratings and the rating generated by the simulation were registered and accounted for.

The simulation was performed separately for the alternatives that were classified and those that were between two viable classes. Thus, it was found that approximately 87.73% of the 34 materials and medicines that had been classified in a single class remained classified in the same initial solutions categories.

**Fig. 4:**
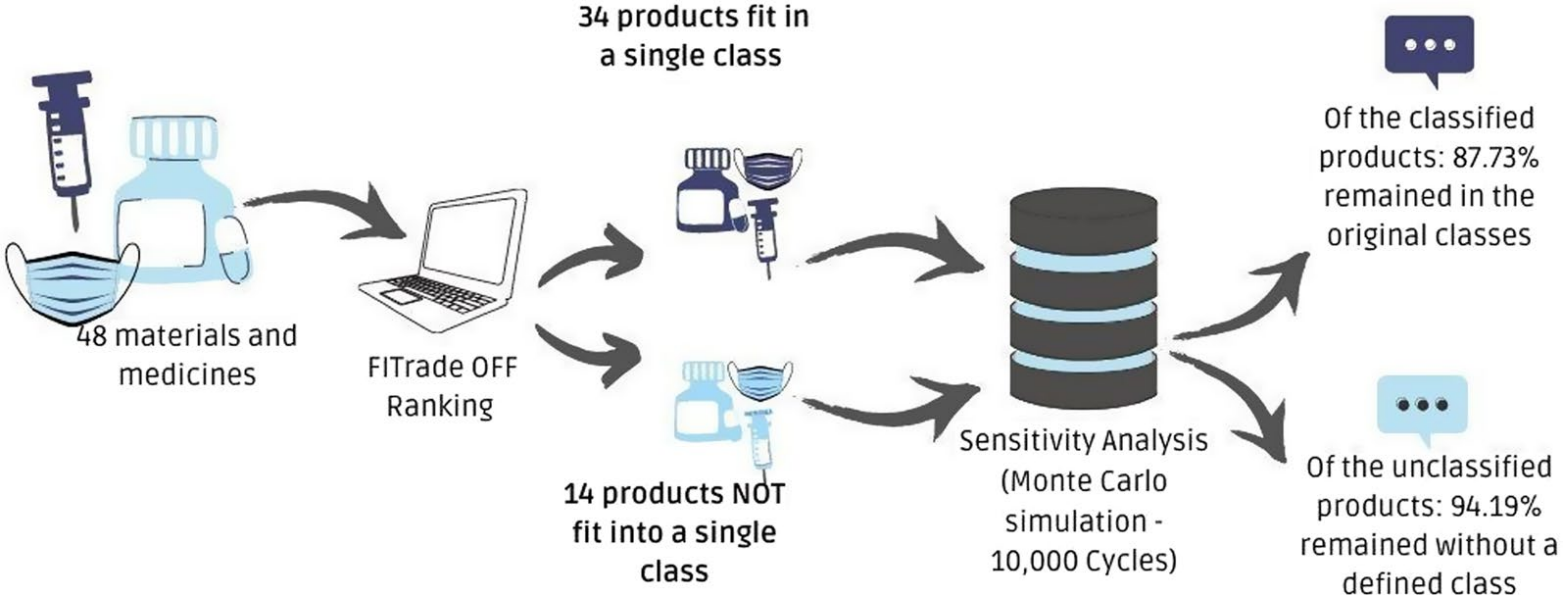
Result synthesis and sensitivity analysis

As for the 14 medicines and materials that were between two viable categories, it was verified that in 94.19% of the 10,000 simulations the result remained the same as the initial one, that is, the alternatives continued between the same two viable classes. The summary of this analysis can be found in Fig. 4.

Once the result analysis was presented to the decision maker and all 48 items were checked to validate that they were placed in the most appropriate class, as per the DM’s experience; the inventory manager validated the allocation of all items in their respective classes.

Although amongst the alternatives selected to solve the problem, only two were in class W, which requires more involvement from the decision maker (inventory manager). It was suggested to the decision maker that as alternatives for this class are added, those responsible for the stock must supervise the parameters that involve these alternatives highlighted in the provision.

Furthermore, when it comes to the alternatives that were between two viable classes, the inventory manager should insert the item in the most critical class, such as the Surgical Trepanning Tool (Table 8) that is between class M and B, with global values of 0.209386 and 0.430647, must be classified in class B, with biweekly monitoring by the inventory manager.

Thereby, the model developed was considered successful in classifying medicines and medical-hospital materials from the HUOL (University Hospital in Brazil) inventory taking into account the decision maker’s preference information. It is relevant to point out that this model is modelled from the reality of a single organization; thus, the criteria and alternatives related in the explored decision problem do not represent the reality of other inventories outside this environment. However, the structuring of the proposed model, taking into account the criteria analysed, may serve as a solid basis for the construction of other similar models, considering the multi-criteria approach and the inventory managers’ preference structure specific for each hospital.

## Conclusion

The current study presented a multi-criteria decision model in hospital management that is rarely addressed in the literature [27–30], therefore the proposed multi-criteria model will allow the allocation of items in 3 classes (W, B M). Items allocated in class W must be monitored weekly. Items allocated in Class B must be monitored biweekly and, finally, items allocated in Class M must be monitored monthly by the inventory manager. In the multi-criteria model’s validation test, it can be seen that from 48 items, only two items were allocated to class W (Table 7) and two items were allocated to classes W and B (Table 8). Therefore, the hospital’s inventory manager should monitor only 4 of the totals of 48 items weekly, leading to a reduction of more than 90% in the need for weekly item monitoring.

As a suggestion for future work, it is recommended that the sorting problematic should be carried out with the largest possible number of items in stock, so that the supply managers have an overview of the importance of the products for the overall inventory management, prioritizing material supervision.

## Data Availability

The datasets used and analysed during the current study are available and included in this published article.
